# Grifonin-1: A Small HIV-1 Entry Inhibitor Derived from the Algal Lectin, Griffithsin

**DOI:** 10.1371/journal.pone.0014360

**Published:** 2010-12-16

**Authors:** Ewa D. Micewicz, Amy L. Cole, Chun-Ling Jung, Hai Luong, Martin L. Phillips, Pratikhya Pratikhya, Shantanu Sharma, Alan J. Waring, Alexander M. Cole, Piotr Ruchala

**Affiliations:** 1 Department of Medicine, David Geffen School of Medicine, University of California at Los Angeles, Los Angeles, California, United States of America; 2 Department of Radiation Oncology, David Geffen School of Medicine, University of California at Los Angeles, Los Angeles, California, United States of America; 3 Department of Molecular Biology and Microbiology, Burnett School of Biomedical Sciences, University of Central Florida College of Medicine, Orlando, Florida, United States of America; 4 Department of Chemistry and Biochemistry, University of California at Los Angeles, Los Angeles, California, United States of America; 5 Materials and Process Simulation Center, California Institute of Technology, Pasadena, California, United States of America; University of California San Francisco, United States of America

## Abstract

**Background:**

Griffithsin, a 121-residue protein isolated from a red algal *Griffithsia sp.*, binds high mannose N-linked glycans of virus surface glycoproteins with extremely high affinity, a property that allows it to prevent the entry of primary isolates and laboratory strains of T- and M-tropic HIV-1. We used the sequence of a portion of griffithsin's sequence as a design template to create smaller peptides with antiviral and carbohydrate-binding properties.

**Methodology/Results:**

The new peptides derived from a trio of homologous β-sheet repeats that comprise the motifs responsible for its biological activity. Our most active antiviral peptide, grifonin-1 (GRFN-1), had an EC_50_ of 190.8±11.0 nM in *in vitro* TZM-bl assays and an EC_50_ of 546.6±66.1 nM in *p24^gag^* antigen release assays. GRFN-1 showed considerable structural plasticity, assuming different conformations in solvents that differed in polarity and hydrophobicity. Higher concentrations of GRFN-1 formed oligomers, based on intermolecular β-sheet interactions. Like its parent protein, GRFN-1 bound viral glycoproteins gp41 and gp120 *via* the N-linked glycans on their surface.

**Conclusion:**

Its substantial antiviral activity and low toxicity *in vitro* suggest that GRFN-1 and/or its derivatives may have therapeutic potential as topical and/or systemic agents directed against HIV-1.

## Introduction

**Figure 1:**
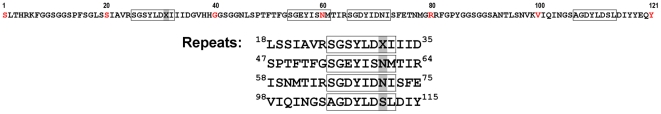
Sequence of the griffithsin and comparison of the functional repeats (boxed) in its structure. Position of the unknown amino acid X^31^ (shaded) is occupied by Ala in the expressed variant of the protein.

Preventing HIV-1 infection is the primary goal of pre- and post-exposure prophylaxis of HIV. Prophylactic modalities that block HIV-1 entry would be particularly valuable, because latent HIV-1 infections are typically refractory to therapeutic or immunological interventions [1]–[9]. Lectins, especially those targeting the high mannose, N-linked glycans of HIV surface glycoproteins [10], are exceptionally potent HIV entry inhibitors. Most anti-HIV lectins have been identified in and isolated from natural sources. They include cyanovirin-N [11], [12] and scytovirin [13] from cyanobacteria, contrajervin and treculavirin from moraceous plants [14], and the θ-defensin peptides of non-human primates [15]–[19]. Among these, the red algal protein griffithsin (GRFT) [20] stands out as having the most potent anti-HIV inhibitory activity, with an average EC_50_ of 40 pM [20], [21]. GRFT has been produced recombinantly in *Escherichia coli* [22], [23] and, in larger quantities, in *Nicotiana benthamiana* plants [24]. GRFT forms homodimers whose binding to high mannose oligosaccharides blocks the binding of gp120 to CD4-expressing cells. Notably, the three almost identical carbohydrate binding sites on each monomer of GRFN [23], [25]–[27] are formed by Tyr and Asp residues in these functional repeats (**Figure 1**).

In addition to high potency [24], GRFT showed stability over a satisfactory pH and temperature range, caused minimal toxicity, and did not induce the release of proinflammatory cytokines that might recruit potential HIV-susceptible target cells to the target mucosa. These desirable properties make GRFT an excellent candidate microbicide [28], as well as an intriguing starting point for the design of smaller peptide-based antiviral minilectins directed against high mannose sugars.

As only two entry inhibitors, Fuzeon® (T20, Enfuvirtide) and Maraviroc, are currently in clinical use, there is a need for new entry inhibitors [29]–[34] that could be used topically to prevent infection, or systemically to treat patients with drug-resistant HIV. Since the systemic use of non-human proteins, including griffithsin or cyanovirin-N, may be time-limited by their immunogenicity, smaller griffithsin-derived peptides may present a more suitable alternative for such applications.

This report describes Grifonin-1 (GRFN-1), an 18-residue peptide with glycan-binding and HIV-inhibitory properties, derived from the β-sheet core of griffithsin.

## Materials and Methods

### Ethics Statement

Venous blood samples (uncoded) were collected in the Department of Medicine at UCLA, by a qualified person under the approved Institutional Review Board protocol (IRB #92-11-596-41) after obtaining written consent from participants.

Primary vaginal epithelial cells (VECs) were purchased from MatTek Corp., (Ashland, MA USA) and provided with no identifiers not allowing direct identification of the donors.

#### Peptide synthesis and characterization

All peptides were synthesized by the solid phase method using a CEM Liberty automatic microwave peptide synthesizer (CEM Corporation, Matthews, NC), applying 9-fluorenylmethyloxycarbonyl (Fmoc) chemistry [35]. Amino acid derivatives and reagents were from EMD Biosciences (San Diego, CA) and Chem-Impex International (Wood Dale, IL). Rink Amide MBHA resin from EMD Biosciences (San Diego, CA) was used as a solid support. Peptides were cleaved from the resin with modified reagent K (TFA 94% (v/v); phenol, 2% (w/v); water, 2% (v/v); TIS, 1% (v/v); EDT, 1% (v/v); 2 hours) and precipitated by adding ice-cold diethyl ether. Reduced peptides were purified by preparative reverse-phase high performance liquid chromatography (RP-HPLC) to >90% homogeneity and their purity evaluated by matrix-assisted laser desorption ionization spectrometry (MALDI-MS) and analytical RP-HPLC.

#### Disulfide bond formation

Peptides were dissolved at a final concentration of 0.25 mg/ml in 50% DMSO in H_2_O and stirred overnight at room temperature. Subsequently, the peptides were lyophilized and re-purified on a preparative C18 SymmetryShield™ RP-HPLC column to >95% homogeneity and their purity was evaluated by MALDI-MS and analytical RP-HPLC.

#### Analytical HPLC

Analytical RP-HPLC was performed on a Varian ProStar 210 HPLC system equipped with ProStar 325 Dual Wavelength UV-Vis detector with the wavelengths set at 220 nm and 280 nm (Varian Inc., Palo Alto, CA). Mobile phases consisted of solvent A, 0.1% TFA in water, and solvent B, 0.1% TFA in acetonitrile. Analyses of peptides were performed with an analytical reversed-phase C18 SymmetryShield™ RP18 column, 4.6×250 mm, 5 µm (Waters Corp., Milford, MA) applying a linear gradient of solvent B, from 0 to 100%, over 100 min with a flow rate of 1 ml/min.

All biological and biophysical experiments presented in the article were performed using unfractionated heterogeneous mixture of oligomers of GRFN peptide(s) (please see Analytical ultracentrifugation section).

#### Griffithsin (GRFT)

Recombinant His-Tagged griffithsin (Cat#11610) was obtained through the AIDS Research & Reference Reagent Program, Division of AIDS, NIAID, NIH.

#### Binding studies

Surface plasmon resonance (SPR) studies were performed on a BIAcore 3000 system (BiaCore AB, Piscataway, NJ), using BIAcore CM5 sensor chips and HBS-EP running buffer (pH = 7.4) containing 10 mM HEPES, 150 mM NaCl, 3 mM EDTA, and 0.005% polysorbate 20. Proteins/peptides were immobilized on a CM5 sensor chip using the amine coupling method. The chip was activated by mixing 400 mM *N*-ethyl-*N*-(3-dimethylaminopropyl)-carbodiimide hydrochloride and 100 mM *N*-hydroxysuccinimide. Residual reactive groups on the chip surface were blocked using 1.0 M ethanolamine/HCl (pH = 8.5). The flow cell-1 chamber, which served as a control, lacked immobilized protein but was treated with *N*-ethyl-*N*-(3-dimethylaminopropyl)-carbodiimide hydrochloride, *N*-hydroxysuccinimide, and ethanolamine/HCl. Binding signals were corrected for nonspecific binding by subtracting the flow cell-1 signal. To regenerate chip surfaces, bound ligands were removed with 10 mM HCl. Data were analyzed with BIAevaluation 4.1 software (Biacore, Piscataway, NJ).

#### Saccharides

α-D-mannopyranose (Man), L-fucopyranose (Fuc), α-D-galactopyranose (Gal), sialic acid (N-acetyl-neuraminic acid, Neu5Ac), N-acetyl-glucosamine (GlcNAc) and Man_3_(GlcNAc)_2_, a core pentasaccharide found in most N-linked oligosaccharides. All of these saccharides were purchased from Sigma-Aldrich (St. Louis, MO).

### Antiviral activity studies

#### Luciferase reporter assay (TZM-bl assay)

[36] The ability of the analogs to inhibit HIV infection was determined with an assay that determines viral infectivity with an LTR-mediated luciferase reporter construct [37]. In brief, TZM-bl reporter cells are HeLa-derived JC53-BL cells that express high levels of CD4, CXCR4, and CCR5. They also contain reporter cassettes for luciferase and β-galactosidase, both driven by the HIV-1 long terminal repeat [38]). 24 hr after these reporter cells were exposed to R5 lab-adapted strains of HIV-1 (BaL) in the presence of peptides, protection was measured as % reduction in luciferase activity (relative light units) compared to cells infected with virus and peptide vehicle alone.

#### p24^gag^ antigen release assay

As previously described [15], immortalized CD4+ lymphoblastic PM1 cells, (1.5×10^5^/100 µl) were incubated with virus (BaL) (MOI = 10^−2^) in the presence or absence of 0.078–5 µM of GRFN-1 for 3 hrs at 37°C/5% CO_2_. The cells were washed and resuspended in fresh growth media containing vehicle or peptides for 7 days. Supernatants were collected at days 3, 5, and 7 post-infection, and on days 3 and 5 the cells were resuspended in 1 ml of growth medium containing the appropriate concentration of vehicle or peptide. HIV-1 infection was quantified by measuring the amount of *p24^gag^* in the cell supernatants (Perkin Elmer p24 ELISA, Waltham, MA). Day 5 supernatants were determined to represent the peak infection day in PM1 cells.

#### Cell viability and cytotoxicity

Cell viability experiments were carried out using CytoTox-Glo™ Assay (Promega Corp., Madison, WI) and cytotoxicity was analyzed using an MTT-based cell proliferation kit (Boehringer Mannheim, Indianapolis, IN) Both tests were performed according to manufacturer's instructions. MTT is 3-(4,5-dimethylthiazol-2-yl)-2,5-diphenyltetrazolium bromide. Experiments utilizing vaginal epithelial cells (VECs) were carried out as previously described [49].

#### Human red blood cells (hRBCs)

Heparinized fresh blood (∼3 ml) was collected from an anonymous donor. A 200 µl aliquot was removed and washed 4× with 800 µl of cold PBS at RT for 3 min at ∼500×g. After each centrifugation, 780 µl of supernatant was removed. After the last wash, the supernatant was completely removed and the packed red blood cells were diluted 1∶20 in PBS to prepare a 5% v/v stock suspension of hRBCs.

#### Primary vaginal epithelial cells

(VECs) were purchased from MatTek Corp., (Ashland, MA USA) and maintained according to provided protocol.

#### Hemolysis assay

As previously described [39], 2.5% v/v normal human RBCs were exposed to various concentrations of GRFN-1 at 37°C for 30 min. All experiments were carried out in triplicate using 96-well microplate (Costar 3596) and the OD at 700 nm was monitored every 30 s. employing the SpectraMAX 250 microplate reader (Molecular Devices, Sunnyvale, CA). Controls included PBS, untreated human RBCs, and human RBCs +2.5% Triton (100% lysis). The hemolytic effect was calculated as follows:
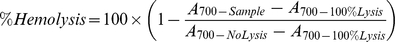



Inflammatory response was assessed using Bio-Plex Human Cytokine Multiplex Assays (Cat# M50-0KCAF0Y & MF0-005KMII, Bio-Rad Laboratories, Inc., Hercules, CA). Briefly, human peripheral blood mononuclear cells and primary vaginal epithelial cells (VECs) were cultured with 0–100 µM of GRFN-1 in appropriate media. After 24 h supernatants were collected and assayed according to manufacturer's protocol. Only data for factors with readable, “in range” values are presented.

#### Circular dichroism (CD) analyses of secondary structure

CD spectra from 185–260 nm of GRFN-1 were examined in different solution environments using a JASCO 720 spectropolarimeter (Jasco, Easton, MD) calibrated for wavelength and optical rotation with 10-camphorsulphonic acid [40], [41]. Peptide was scanned at 20 nm per minute in a 0.01 cm path-length cell at 25°C with a sample interval of 0.2 nm. The peptide concentration was determined by UV absorbance at 280 nm. After baseline correction, spectra were expressed as the Mean Residue Ellipticity [θ]_MRE_. Quantitative estimates of secondary structural contributions were made with Selcon [42] using the spectral basis set for proteins in the Olis Global Works™ software package (Olis Inc., Bogart, GA).

#### Fourier transform infrared (FTIR) spectroscopy

Infrared spectra were recorded at 25°C using a Bruker Vector 22™ FTIR spectrometer with a deuterated triglycine sulfate (DTGS) detector, and averaged over 256 scans at a gain of 4 with a resolution of 2 cm^−1^. Peptide samples were initially freeze-dried several times from 10 mM HCl in D_2_O to remove any interfering counter ions and residual H_2_O. Solution spectra of peptides were made in deuterated 10 mM phosphate buffer, pD = 7.4 (pD  =  pH+0.4) and in structure promoting mixed solvent-buffer solutions (trifluoroethanol (TFE) or hexafluoroiso propanol (HFIP). Spectra were acquired using a temperature controlled, demountable liquid cell with calcium fluoride windows fitted with a 50 µm thick spacer (Harrick Scientific, Pleasantville, NY). The relative proportions of α-helix, β-turn, β-sheet, and disordered conformations of solution and multilayer IR spectra were determined by Fourier self-deconvolution for band narrowing and area calculations of component peaks of the FTIR spectra using curve-fitting software supplied by Galactic Software (GRAMS/AI, version 8.0; Thermo Electron Corp., Waltham, MA). The frequency limits for the different structures were: α-helix (1662–1645 cm^−1^), β-sheet (1637–1613 and 1710–1682 cm^−1^), turns (1682–1662 cm^−1^), and disordered or random (1650–1637 cm^−1^) [43].

#### Molecular dynamics modeling

The starting structure for GRFN-1 was obtained by using Hyperchem 7.5 (http://www.hyper.com) to template the peptide structure in a beta hairpin conformation derived from segments of the griffithsin protein (PDB 2GTY, residues 18–31). These monomeric starting structures were placed in a periodic 56Å^3^ box of TIP4P water and the ensemble was neutralized with counter ions to simulate the environment used for the experimental CD measurements. The peptide in the solution box was conjugate-gradient minimized using the Polak-Ribiere approach implemented in Hyperchem. The minimized monomeric GRFN-1 ensemble was ported to the Gromacs program suite, version 4.0.4 (http://www.gromacs.org), and subjected to the steepest descent method using the OPLS_AA option [44].

The system was subjected to 20 psec of pre-run molecular dynamics at 300°K allowing the solvent to equilibrate while restraining the peptide. After pre-run solvent equilibration, the peptides were subjected to 50 nsec of free MD simulations at 300°K without any experimental constraints, utilizing Berendsen temperature and pressure coupling and the Particle Mesh Ewald method for evaluating long-range electrostatic interactions. The time-dependent evolution of the peptide secondary structure (i.e., analyzed using the DSSP criteria [45] for the peptide in the water environment indicated when equilibrium was reached. Molecular model illustrations were rendered using PyMOL v0.99 (http://www.pymol.org).

#### Analytical ultracentrifugation

Sedimentation equilibrium was performed at 20°C in a Beckman Optima XL-A analytical ultracentrifuge using absorption optics at 280 nm. Twelve mm path length double sector cells were used for all samples. Samples (OD_280_ = 0.7 and OD_280_ = 0.15)) were in 20 mM NaH_2_PO_4_. Sedimentation equilibrium profiles were measured at speeds of 3000, 7000, 11000, 24000 and 36000 rpm. The data were fitted with a nonlinear least-squares exponential fit for a single ideal species using the Beckman Origin-based software (Version 3.01 A partial specific volume of 0.739 was estimated from the amino acid composition [46] using 0.9 (the partial specific volume of a leucine residue) for the partial specific volume of cyclohexylalanine and cyclohexylglycine. It was then corrected to 20°C [47].

## Results

### Design and synthesis of Grifonins (GRFNs)

The remarkable reported activity of griffithsin against laboratory strains and primary isolates of T- and M-tropic HIV-1 prompted us to perform a detailed structural analysis of this protein as the first stage of our attempt to design small peptidic analogs that potentially could mimic the anti-viral properties of the native griffithsin.

Structurally, three homologous β-sheet domains (residues 20–34, 58–76 and 96–120) form the protein's core (**Figures 1** **&** **2**) and contain the functional repeats responsible for its carbohydrate-binding [23]. Since the protein lacks disulfide bonds, its integrity is most likely maintained by hydrophobic and ionic interactions. We theorized that each β-sheet domain can either act independently as an entry inhibitor and/or assemble into the higher order structures resembling those of native griffithsin. Consequently, some of our peptide analogues contained unusual amino acids and/or structural features not present in native griffithsin to impose secondary structural features, which we hypothesized might be important for activity and multimerization. Specifically, we introduced varying numbers of cyclohexylalanine (Cha) and cyclohexylglycine (Chg) residues that might “dial-up” hydrophobic interactions between monomers and possibly increase assembly of higher order structures (desirably trimers). Some analogs also contained a disulfide bridge to stabilize β-hairpin-monomer structure. After re-evaluating the initial group of compounds with respect to their composition, hydrophobicity and ease of synthesis, we concluded that 3 analogues shown in **Table 1** were feasible for further development. An attempt to obtain folded (oxidized) GRFN-1 directly on the solid support using thalium trifluoroacetate ((CF_3_COO)_3_Tl) protocol [48] was unsuccessful. Consequently, after solid-phase synthesis, the linear peptides were oxidized in 50% DMSO to form the desired β-hairpin GRFNs. Analytical data for synthesized peptides are presented in **Table 2** & **Figure 3**.

**Figure 2:**
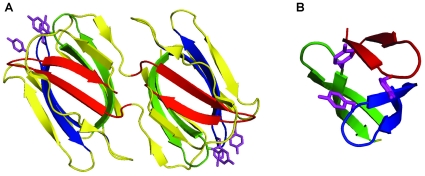
The structure of griffithsin. Panel A: Dimer of griffithsin (PDB entry code 2GTY, [23]). Fragments corresponding to GRFN-1/2 and GRFN-3 are in red and in blue respectively. Remaining homological domain necessary to form core of the griffithsin's monomer is in green. Panel B: Core of griffithsin. Residues Tyr^28^, Tyr^68^ and Tyr^110^, which are components of monosaccharides' binding domain, are in magenta.

**Figure 3:**
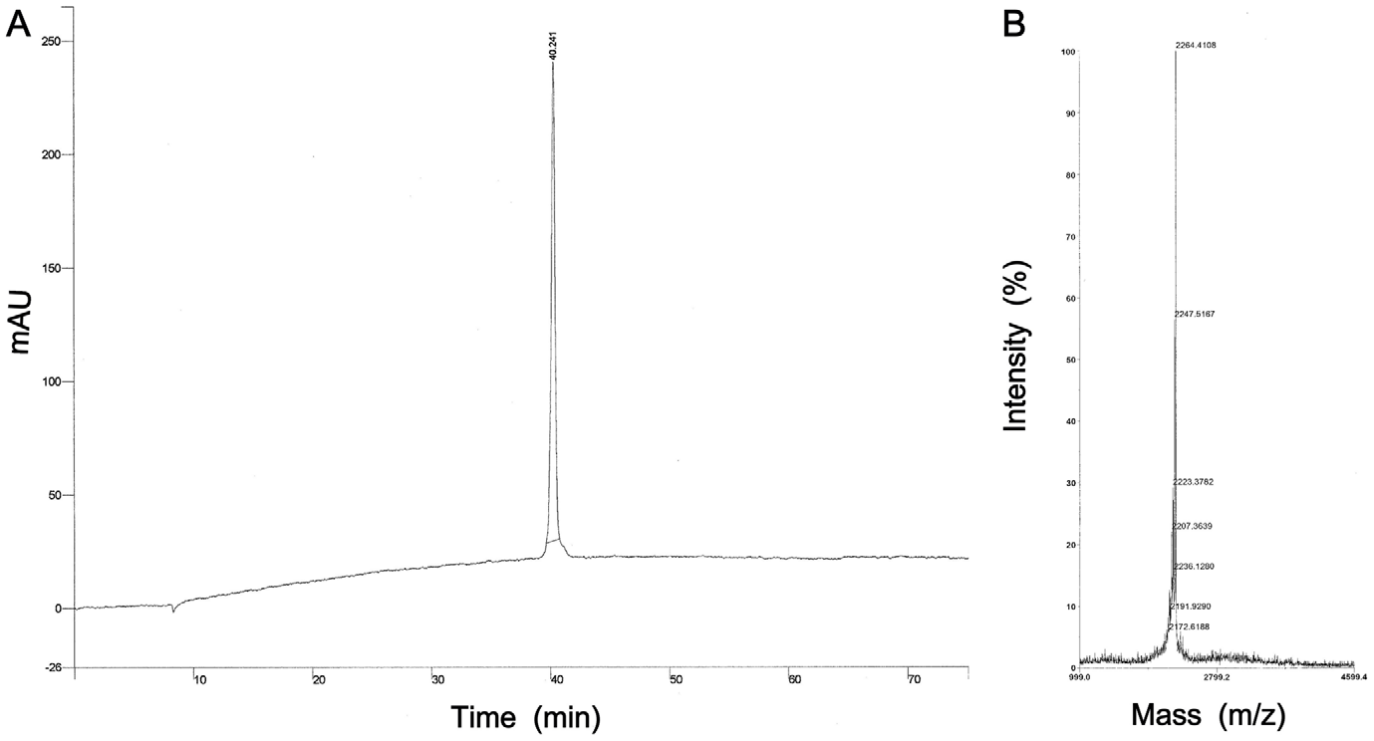
Analytical data for GRFN-1. (A) analytical HPLC profile and (B) MS spectra.

### Antiviral activity of GRFNs

**Table 1:** Sequences of Grifonins (GRFNs).

Peptide	Sequence
GRFN-1	Cha-SC-Chg-R-Chg-RSGSY-Cha-DN-Chg-R-Chg-(D)Cys-CONH_2_
GRFN-2	Cha-SC-Chg-R-Chg-RSGSY-Cha-DR-Ch\g-R-Chg-(D)Cys-CONH_2_
GRFN-3	Chg-CR-Chg-R-Chg-RSGDY-Chg-DR-Chg-R-Cha-(L)Arg-(D)Cys-CONH_2_

Cysteins forming intra-molecular disulfide bonds are bolded. *Cha-(L)-Cyclohexyl-alanine, Chg-(L)-Cyclohexyl-glycine, (D)Cys-(D)-Cysteine.*

**Table 2:** Analytical data of GRFN peptides.

Peptide	Composition	MW (g/mole) Calc/Found	R_T_ (min)
GRFN-1	C_102_H_167_N_29_O_25_S_2_	2263.78/2264.41	40.241
GRFN-2	C_104_H_173_N_31_O_24_S_2_	2305.86/2306.61	39.100
GRFN-3	C_113_H_190_N_38_O_25_S_2_	2545.14/2546.12	32.602

R_T_- retention time.

**Figure 4:**
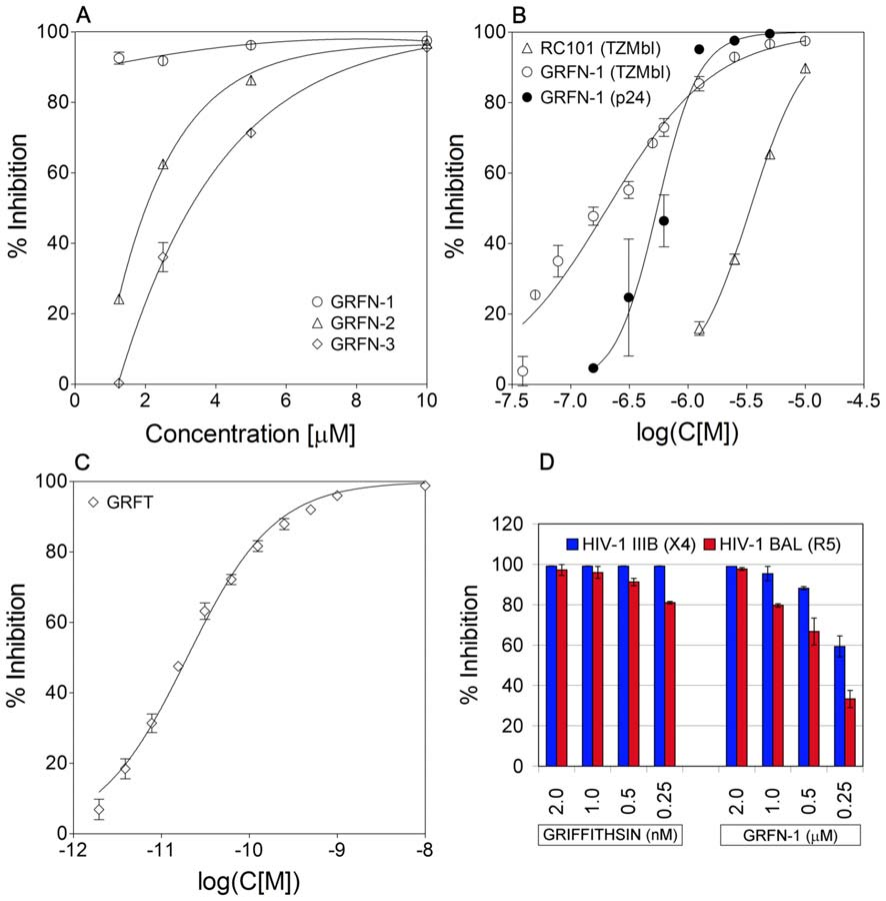
Antiviral activity of GRFNs. Panel A: Comparison of dose response experiments of GRFNs in TZM-bl assay. Panel B: Comparison of dose response experiments of GRFN-1 in TZM-bl assay (EC_50_ = 190.8±11.0 nM) and *p24^gag^* antigen release assay (EC_50_ = 546.6±66.1 nM) with RC-101 in TZM-bl assay (EC_50_ = 3404.0±91 nM). RC-101 is a θ-defensin which is currently being developed as topical microbicide. Panel C: Antiviral activity of griffithsin (GRFT) in TZM-bl assay (EC_50_ = 19.6±1.9 pM). Panel D: Comparison of antiviral activity of GRFT and GRFN-1 in *p24^gag^* antigen release assay using PBMCs and CXCR4 (HIV-1_IIIB_) and CCR5 (HIV-1_BAL_) strains.

Initially, grifonins 1–3 were screened in a standard HIV-1 reporter assay (**Figure 4A**). Dose response experiments revealed that GRFN-1 was the most potent analog and that it maintains high activity at low µM concentrations. Consequently we chose GRFN-1 as our lead compound and re-tested a broader range of concentrations in the TZM-bl assay as well as in the more stringent *p24^gag^* antigen release assay using immortalized CD4+ lymphoblastic PM1 cells (**Figure 4B**). This 18 amino acid long peptide showed considerable activity in both assays with EC_50_ values 190.8±11.0 nM and 546.6±66.1 nM for TZM-bl and *p24^gag^* antigen release assays respectively. GRFN-1 was also more potent than retrocyclin (RC)-101, which had an EC_50_ of 3,404±91 nM in the TZM-bl assay. RC-101, which served as positive control, is a humanized θ-defensin, which is being developed as a prospective topical microbicide [49]. However, GRFN-1 was significantly less active than the parental protein griffithsin (**Figure 4C**) that in our hands showed potent activity in low picomolar range (EC_50_ = 19.6±1.9 pM). Both, griffithsin and GRFN-1 maintained their antiviral activity in PBMC based *p24^gag^* antigen release assays toward CXCR4 (HIV-1_IIIB_) as well as CCR5 (HIV-1_BAL_) strains (**Figure 4D**).

### GRFN-1 binds viral glycoproteins

**Figure 5:**
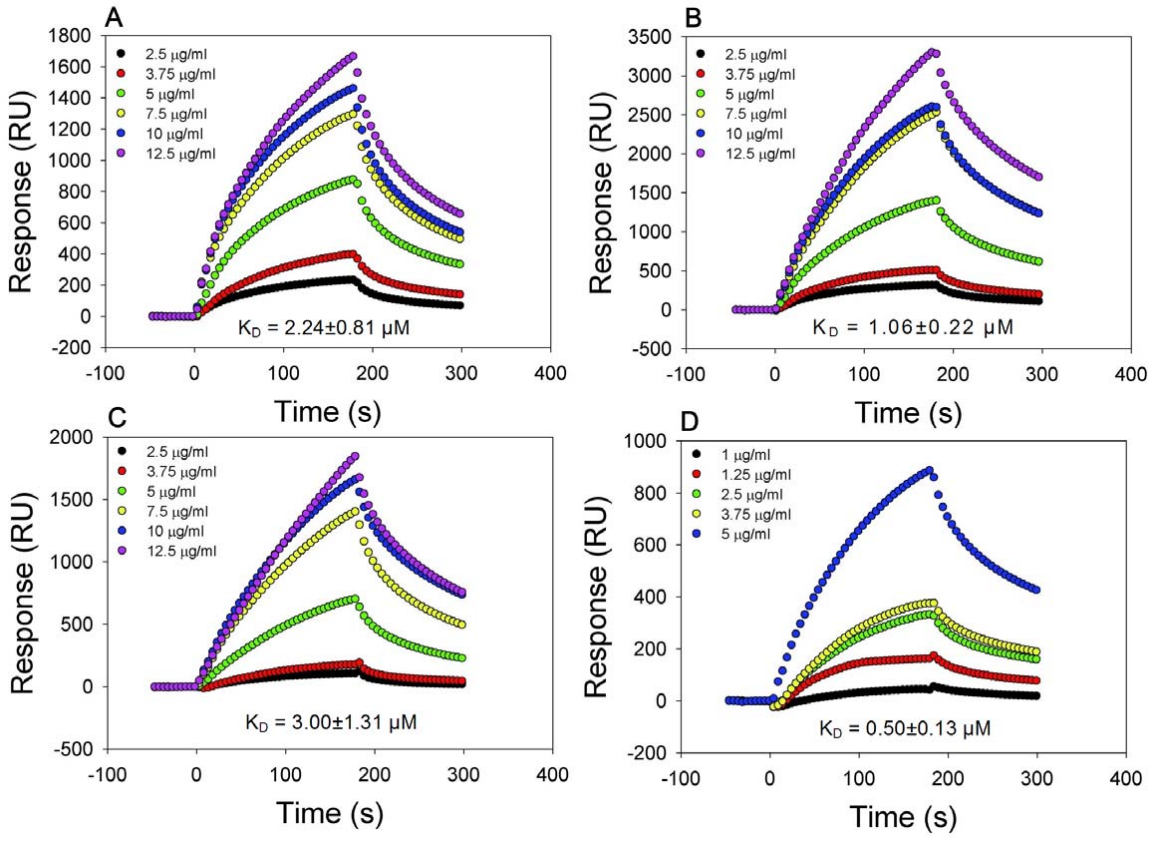
Binding of GRFN-1 to viral glycoproteins. Binding to: (A) gp120_LAV_, (B) gp120_BAL_, (C) gp41 and (D) GRFN-1 (self-association). K_D_±SEM values were calculated as an average from at least 5 independent experiments.

As N-linked glycans are the molecular target(s) of griffithsin, we sought to determine whether GRFN-1 acts *via* similar mechanism. To ascertain this, we performed two types of surface plasmon resonance (SPR) binding experiments. In the first set, we established that GRFN-1 binds gp41, gp120_BAL_ and gp120_LAV_ in a dose dependent manner (**Figure 5A–C**) with K_D_ values in low micromolar range (K_D_ = 1.06±0.22 to 3.00±1.31 µM). **Figure 5D** shows that the K_D_ (0.50±0.13 µM) for the self-association of GRFN-1 was considerably below its K_D_ for binding to the aforementioned viral glycoproteins. This finding suggested that GRFN-1 acts as a multimer rather than as a monomer.

**Figure 6:**
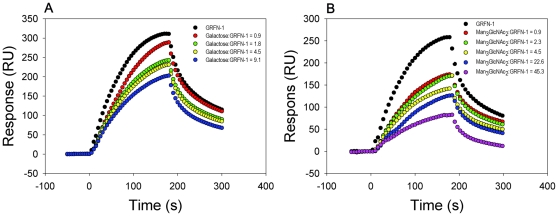
Examples of SPR competition experiments of GRFN-1 with saccharide(s) using gp120_LAV_ chip. (A)- galactose, (B) Man_3_GlcNAc_2_ (“core pentasaccharide”).

Binding to N-linked glycans was additionally confirmed in competition experiments employing various saccharide components of such glycans: mannose (α-D-mannopyranose, Man), fucose (L-fucopyranose, Fuc), galactose (α-D-galactopyranose Gal), sialic acid (N-acetyl-neuraminic acid, Neu5Ac), N-acetyl-glucosamine (GlcNAc) and common core pentasaccharide Man_3_GlcNAc_2_ (**Figure 6** **& Supporting Information S1**). Although GRFN-1 interacted with all of these sugars, except fucose, in the range of concentrations tested, the most effective competitors were GlcNAc and mannose.

### Cytotoxicity and pro-inflammatory properties

**Figure 7:**
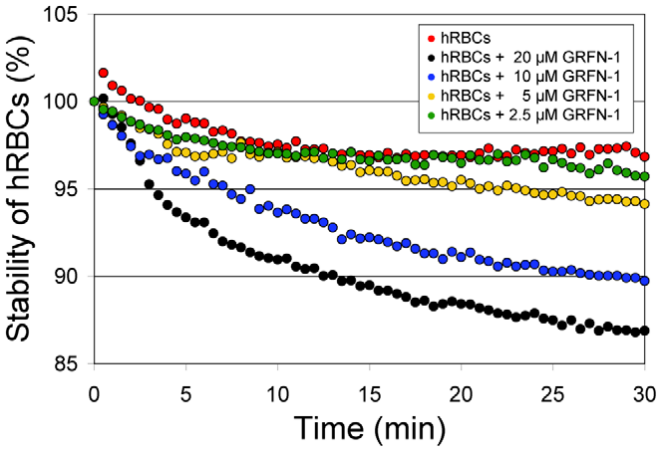
Stability of human RBCs in the presence of various concentrations of GRFN-1.

**Figure 8:**
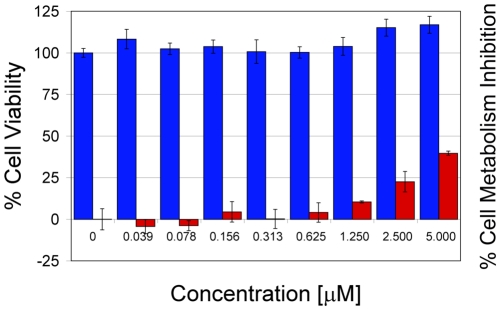
Viability (blue) and % of cell metabolism inhibition (red) of TZM-bl cells in the presence of various concentrations of GRFN-1.

**Figure 9:**
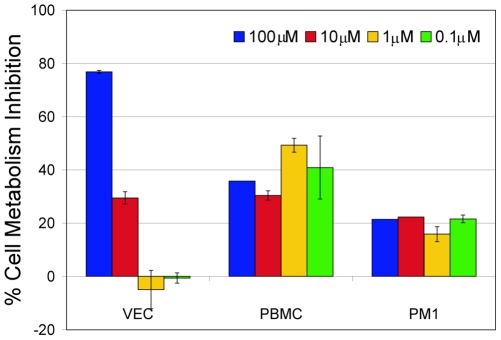
Inhibition of cell metabolism by various concentrations of GRFN-1 (MTT assay) determined in primary vaginal epithelial cells (VEC), human peripheral blood mononuclear cells (PBMC) and PM1 cells (continuously CD4+ T-cell line, [**52**]).

Since systemic uses would typically require either intravenous or subcutaneous administration of the GRFN-1, we performed hemolysis assays using various concentrations of the peptide and human red blood cells (hRBCs) (**Table 3** **&** **Figure 7**). The compound showed low hemolytic activity (∼10%) toward hRBC at the highest concentration tested (20 µM) and was not hemolytic at concentrations below 2.5 µM. Toxicity studies of GRFN-1 with TZM-bl cells (**Figure 8**) showed no effect on viability, although the peptide appeared to inhibit cellular dehydrogenase activity (as gauged by MTT reduction) in a dose dependent manner. Studies with primary vaginal epithelial cells (VEC), peripheral blood mononuclear cells and PM1 cells showed similar effects (**Figure 9**) although actual level of metabolic inhibition was strongly dependent on cell type.

**Table 3:** Hemolytic effect of GRFN-1 on human red blood cells (hRBCs).

Concentration (µM)	Hemolysis±SEM (%)
20	10.3±4.3
10	7.3±1.3
5	2.8±2.3
2.5	1.2±0.7
≤1.25	Non hemolytic

One of the important properties of antivirals is lack of pro-inflammatory properties. We tested GRFN-1 for such properties using human PBMCs and primary VECs. Cells were treated with the peptide for 24 hr and subsequently supernatant was analyzed using Bio-Plex Human Cytokine Multiplex Assays (Bio-Rad Laboratories, Inc., Hercules, CA) for various pro-inflammatory cytokines and growth factors. Results are presented in **Supporting Information S1**. We found a number of factors to be either unchanged or decreased after treatment with GRFN-1. These include IL-5, IL-8, IL-10, IL-13, VEGF, IFN-γ, TNF-β, GM-CSF, MIP-1α and others. Notably, reverse effect of GRFN-1 treatment was also observed for limited number of pro-inflammatory factors.

### Secondary structure analysis of GRFN-1

#### Analysis by FTIR spectroscopy

**Figure 10:**
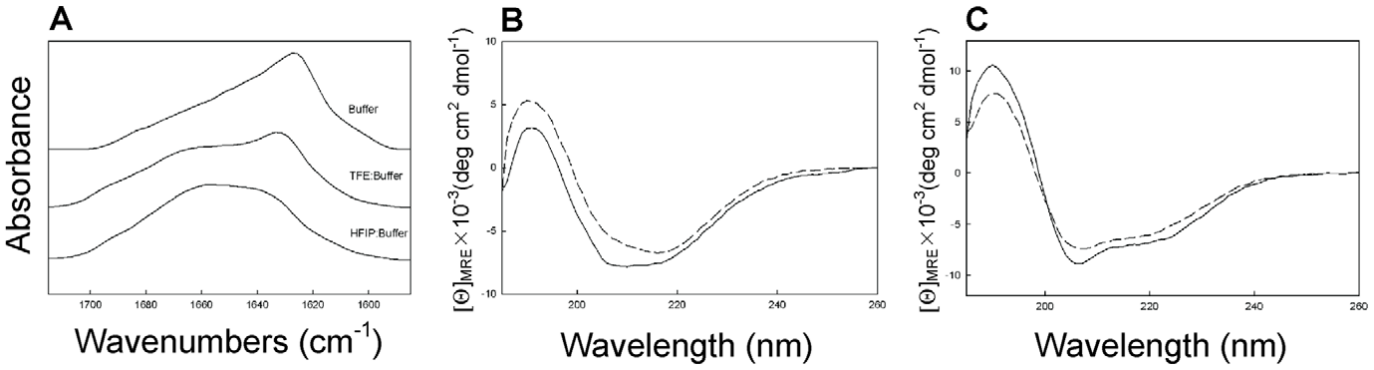
Analysis of GRFN-1 structure. (A) FTIR spectra; (B) CD spectra in in 10 mM phosphate buffer, pH = 6.5 ((---) 0.25 mM; (─) 0.5 mM); (C) CD spectra in TFE:10 mM phosphate buffer pH = 6.5 (4∶6, v∶v) and HFIP (---); 10 mM phosphate buffer pH = 6.5 (4∶6, v∶v) (─).

Analysis of the secondary structure of GRFN-1 in solvent systems of varying polarity are shown in **Figure 10A**. In aqueous buffer the peptide has a dominant β-sheet structure (**Table 4**). In less polar environments such as the amphipathic TFE:buffer solvent system and the more hydrophobic HFIP:buffer environment, there was a shift from β-sheet to more helical conformations with greatest helical propensity in the more hydrophobic environment (**Table 4**). These observations suggest that the GRFN-1 peptide can assume different conformations, depending on the polarity of the solvent.

**Table 4:** Proportions of different elements of secondary structure for GRFN-1 peptide in aqueous buffer, TFE-buffer and HFIP-buffer based on FTIR spectroscopic analysis.

Sample *	Conformation (%)			
	α-helix	β-sheet	turns	disordered
GRFN-1, 0.5 mM in Buffer	12.4	37.6	22.4	27.6
GRFN-1, 0.5 mM in TFE: Buffer	23.2	31.7	20.3	24.8
GRFN-1, 0.5 mM in HFIP:Buffer	48.6	8.4	17.2	25.8

*peptides in 10 mM phosphate buffer pH = 7.5, TFE:10 mM phosphate buffer pH = 7.5 (4∶6, v∶v) or HFIP:10 mM phosphate buffer pH = 7.5 (4∶6, v∶v) were analyzed for secondary conformation based on secondary structural analysis using GRAMS/AI (Methods).

#### Analysis by CD spectroscopy

The conformational plasticity of GRFN-1 was further studied by CD spectroscopy. In 10 mM phosphate buffer, pH = 6.5, GRFN-1 shows a concentration dependent change in secondary structure (**Figure 10B**). At 250 µM, almost equal proportions of α-helix, β-sheet, turn and disordered conformations are present. However, at 500 µM the β-sheet structure increases at the expense of α-helix (**Table 5**). In contrast to the concentration-dependent secondary structural changes in aqueous buffer, the conformation of GRFN-1 in structure-promoting solvent systems such as TFE:buffer and HFIP:buffer (**Figure 10C**) had dichroic minima at 222 and 208 nm with a maximum near 193 nm. These features, characteristic of peptides with more helical conformations [50], were not concentration dependent over the range of 50 to 500 µM (data not shown). Analysis of the CD spectra with curve-fitting algorithms (**Table 5**) reveal that GRFN-1 had a dominant helical conformation in HFIP:buffer and a mix of α-helix, β-sheet, turns in TFE:buffer suggesting that the peptide has a polarity dependent polymorphism.

**Table 5:** Proportions of different elements of secondary structure for GRFN-1 peptide in aqueous buffer, TFE-buffer and HFIP-buffer based on Circular Dichroic spectroscopic analysis.

Sample *	Conformation (%)			
	α-helix	β-sheet	turns	disordered
GRFN-1, 0.25 mM in Buffer	25.0	24.0	21.0	30.0
GRFN-1, 0.5 mM in Buffer	13.0	35.0	21.0	31.0
GRFN-1, 0.5 mM in TFE: Buffer	19.0	28.0	23.0	30.0
GRFN-1, 0.5 mM in HFIP:Buffer	52.0	8.0	16.0	24.0

*peptides in 10 mM phosphate buffer pH = 6.5, TFE:10 mM phosphate buffer pH = 6.5 (4∶6, v∶v) or HFIP:10 mM phosphate buffer pH = 6.5 (4∶6, v∶v) were analyzed for secondary conformation based on secondary structural analysis using Selcon (Methods).

### Molecular dynamics simulations

**Figure 11:**
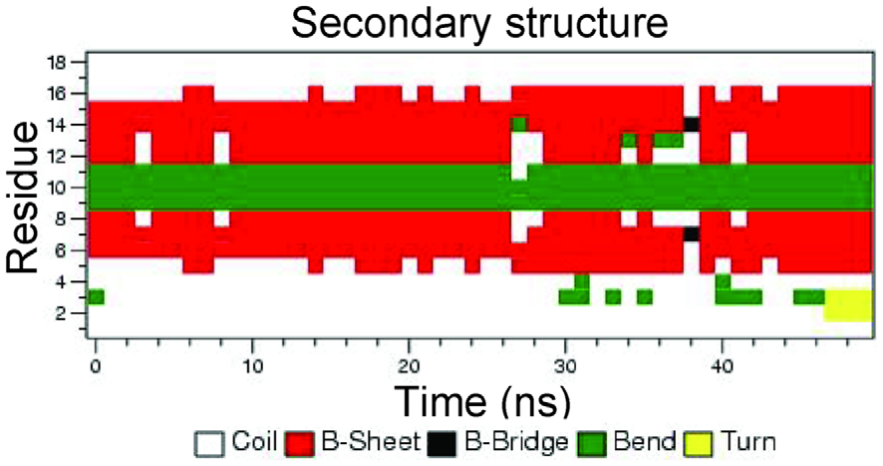
Evolution of GRFN-1 secondary structure as a function of simulation time in aqueous periodic solvent box.

**Figure 12:**
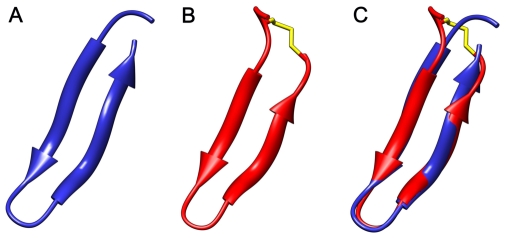
Comparison of GRFN-1 and corresponding griffithsin fragment structures. (A) residues 18–35 of griffithsin in blue (PDB 2GTY), (B) structure of GRFN-1 in red and their overlay (C). Structure of GRFN-1 was obtained from molecular dynamics simulation in water for 50 ns.

To further investigate similarities between their structural elements, molecular dynamics simulations were performed using the conformation of griffithsin residues 18–31 as a homology template for the starting structure of GRFN-1. The templated structure was then subjected to 50 nsec of dynamics without constraints to refine the structure. The DSSP plot (**Figure 11**) indicates that the peptide assumes a stable secondary structure with well defined loop and β-sheet segments. This disposition of secondary structure in the peptide construct is very similar to that observed in the parent griffithsin crystal structure (**Figure 12**) and is consistent with the type and amount of secondary structure observed experimentally in the FTIR and CD measurements of the GRFN-1 peptide in aqueous medium.

### Analytical ultracentrifugation

Sedimentation equilibrium suggests that GRFN-1 in diluted salt is present as high molecular weight soluble aggregates with some monomer present. When initially dissolved in 20 mM NaH_2_PO_4_ the peptide gave a slightly turbid solution. Very little (less than 5%) of the OD_280_ was lost on centrifugation at 3000 rpm. Early scans (after 16 hours of centrifugation) suggested the sample was quite heterogeneous, with a weight-average molecular weight of about 150,000. The samples were then examined at higher speeds. Approximately two-thirds of the OD_280_ was lost at 7000 rpm, with the remaining material being heterogeneous (as seen by the non-random residuals from a single-exponential fit visible in **Supporting Information S1** with a weight-average molecular weight of about 69000. Further scans at 11000, 24,000 and 36,000 rpm gave successively lower weight-average molecular weights as the higher molecular weight complexes were removed by centrifugation (**Table 6**). By 36,000 rpm the remaining material (approximately one quarter of the original OD) had a weight-average molecular weight, 2200, and random residuals from a single-exponential fit consistent with the remaining material being reasonably homogeneous monomer.

**Table 6:** Analytical ultracentrifugation results.

Speed	Molecular weight range (Da)
3K (limited data)	∼150000
7K	68500–84700
11K	8950–38100
24K	2541–6200
36K	2245–2940

## Discussion

Although 30 years of concerted research have led to impressive progress in the therapy of patients infected with HIV-1, therapy remains imperfect and chemoprevention of HIV infections remains an unmet challenge. In this report we present data for a novel HIV-1 entry inhibitor, grifonin-1 (GRFN-1), that was obtained by modifying and truncating the naturally occurring lectin, griffithsin. GRFN-1 peptide is over 6 times smaller than the original protein (18 residues vs. 121), and it is only half the size of Fuzeon® (18 vs. 36 residues), a peptidic entry inhibitor in clinical use. These features make GRFN-1 an attractive compound for further development.

We initially synthesized 3 closely related analogues (GRFNs 1–3) that were engineered to form stable β-hairpin structures that simulated structural features found in the native protein. The properties of each monomer were further modified to enhance self-assembly into higher order structure(s) by increasing hydrophobicity at certain positions (Chg/Cha modifications). Our most active antiviral peptide, GRFN-1, contained a largely intact loop region (–^24^RSGSYLDN^31^–) modified only by the chemically conservative substitution of Leu by cyclohexylalanine. The Asn^31/14^ residue appeared to be crucial for antiviral activity in low concentrations since GRFN-2, an otherwise identical peptide with an Asn to Arg substitution (**Figure 4A**) was considerably less active than GRFN-1. Asn^31^ is not implicated in interactions responsible for ligand(s) binding [23], [25]–[27], neither it is responsible for interactions between functional domains. Nonetheless, in GRFN-1 it may promote oligomerization since the Asn side chain may serve as both a donor and acceptor of hydrogen bonds [51]. Theoretically, inserting a positively charged Arg in the same position could create a repulsive force based on electrostatic interactions.

Oligomerization of GRFN-1 in various solvents (water, DMSO and their mixtures) was suggested in our limited NMR studies (data not shown). Therefore we decided to research this phenomenon in a greater detail. SDS-PAGE analysis in non-reductive conditions was inconclusive (**Supporting Information S1**), however it suggested that dimers of GRFN-1 (∼4.5 kDa) may be stable enough to persist in this relatively “hostile” environment. In addition, SPR experiments demonstrated that GRFN-1 can self-associate at low micromolar concentrations, (K_D_ = 0.50±0.13 µM) strengthening the likelihood that GRFN-1 oligomers contribute substantially to the activities demonstrated in our antiviral assays.

Analytical ultracentrifugation experiments provided insight into the size distribution of GRFN-1 oligomers and/or aggregates, showing that GRFN-1 forms ensembles that range in size from 2.2 (monomer)−150 kDa (**Table 6**). Whether these higher order aggregates of GRFN-1 form any sort of regular structure(s) is difficult to predict from our data, however a “multimer-based” mode of action in *in vitro/in vivo* settings is highly probable. This may also account for the relatively high biological activity of GRFN-1, considering that the potency of its parental molecule, griffithsin, seems to be strongly associated with its multivalency [25]. Indeed, native griffithsin forms a domain swapped dimer with three almost identical carbohydrate-binding sites in each monomer. Such a mode of action may be advantageous since multivalent aggregates are likely to form more stable complexes with viral glycoproteins. In addition, they may be more resistant to proteolysis and more persistent in the bloodstream due to size imposed delay in renal excretion. Notably, based on EC_50_ obtained in various antiviral assays, our leading compound (GRFN-1) is approximately 100 to 1000 times less effective than the parental protein griffithsin. Such a result might be explained by formation of imperfect oligomers that only partially mimic spatial arrangement of griffithsin dimer and its carbohydrate binding centers. In addition, GRFN-1 forms various size oligomers as illustrated by our ultracentrifugation studies, that is in contrast with very stable dimers formed by griffithsin. Similarly, stability of the GRFN-1 oligomers might also impose a detrimental effect on biological activity, since self-association of the peptide is rather moderate (K_D_  = 0.50±0.13 µM) and complexes may not be “stable enough” leading to substantially lower biological activity outcome.

From our SPR binding studies, GRFN-1 like its parental molecule, likely acts by binding N-linked carbohydrates on viral glycoproteins gp41 and gp120. However, competition experiments revealed certain binding preferences (**Supporting Information S1**). Although each of the sugars that we tested, except fucose, competed with the bindin of GRFN-1 to viral glycans, the inhibitory effects depended on the carbohydrate's structure and concentration. Inhibition of binding by GlcNAc and Gal was immediate and “saturated” at a molar ratio of ∼4∶1. D-Mannose was an especially potent inhibitor that could almost completely abrogate binding of GRFN-1 to the viral glycoproteins. Inhibition by sialic acid (Neu5Ac) was less profound and analysis was complicated by the accumulation of sialic acid, most likely due to ionic interactions with the peptide.

**Figure 13:**
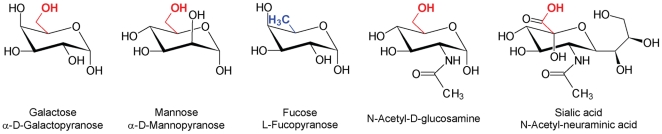
Monomeric components of N-linked glycans. All depicted carbohydrates were used in SPR competition studies. Critical hydroxymethyl moiety is in red and methyl group in position 5 of fucose is in blue.

Given the substantial similarity between the tested carbohydrates, it is interesting to speculate why only fucose failed to compete against GRFN-1's binding to viral glycoproteins. The most striking structural difference between fucose and the other group members is absence of an equatorial hydroxymethyl (–CH_2_OH, in red in **Figure 13**)) in position 5 of the pyranose ring, which is occupied by a methyl group in fucose. This small difference seems to be pivotal for binding by GRFN-1. It is noteworthy that crystallographic analyses of complexes between griffithsin and various carbohydrates [23], [26] demonstrated that the same hydroxymethyl group plays an important role in the hydrogen bond network that underlies its carbohydrate interactions- a finding that also underlines mechanistic similarities between grifithsin and its peptide derivative, GRFN-1.

The presented study identifies a novel 18-residue peptide, GRFN-1, that manifests potent anti-HIV-1 activity. Its low toxicity, limited hemolytic/proinflammatory properties, activity against CCR5 and CXCR4 HIV-1 strains and relatively small size identifies it as a strong lead candidate for further development as HIV-1 entry inhibitor for topical or systemic applications.

## Supporting Information

Supporting Information S1. With Figures S1, S2, and S3, and Tables S1 and S2.
(3.70 MB PDF)
