# Supplementary material for: Grifonin-1: A Small HIV-1 Entry Inhibitor Derived from the Algal Lectin, Griffithsin
Source: PLoS One. 2010 Dec 16;5(12):e14360. doi: 10.1371/journal.pone.0014360 (PMC3002932; doi:10.1371/journal.pone.0014360)
Supplement: Supporting Information S1. — With Figures S1, S2, and S3, and Tables S1 and S2. (3.70 MB PDF) [file pone.0014360.s001.pdf]

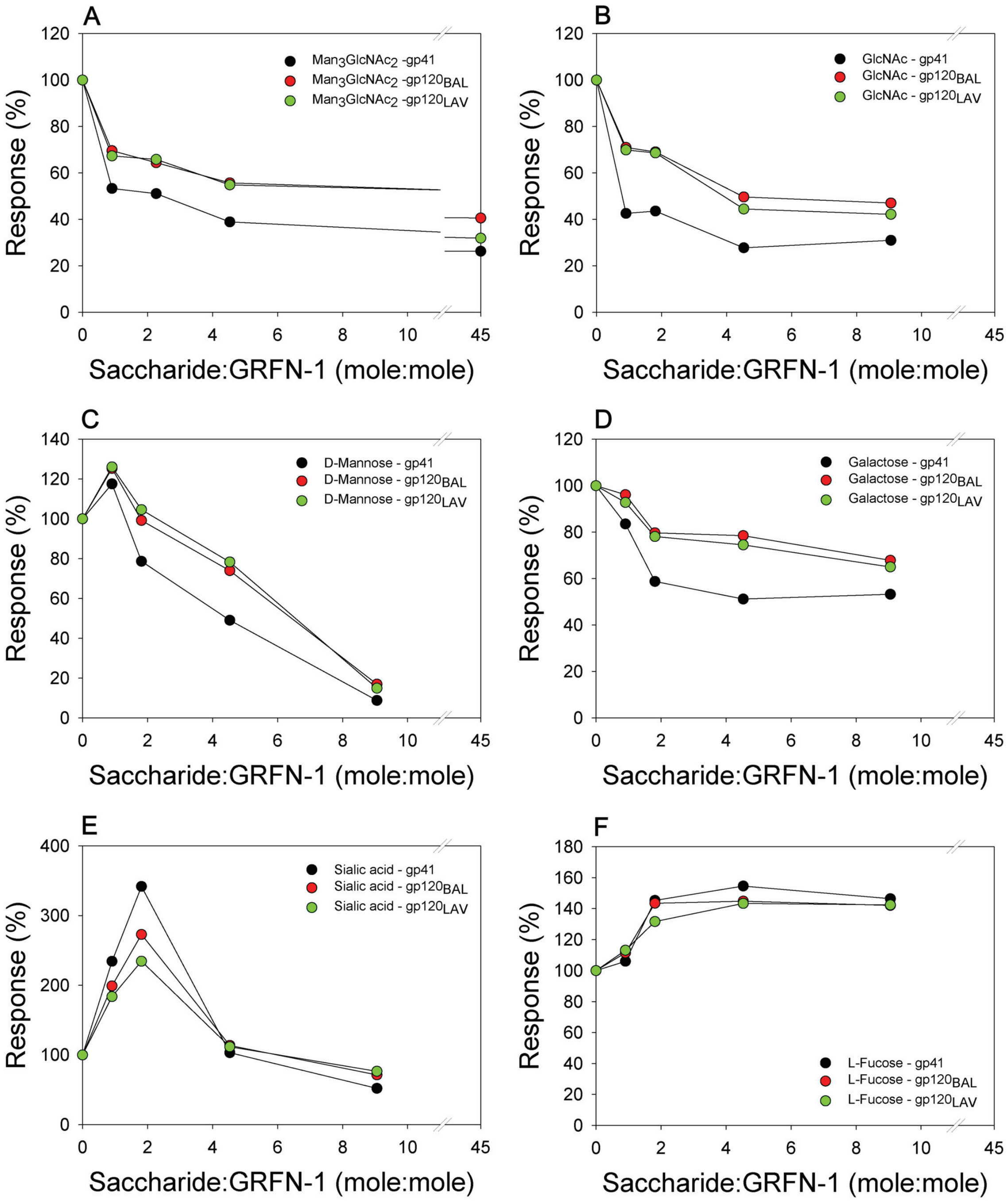

Figure S1. SPR competition experiments of GRFN-1 with various components of N-linked glycans using immobilized gp41, $gp120_{BAL}$ and $gp120_{LAV}$. Competing saccharides: A-$Man_3GlcNAc_2$ ("core pentasaccharide"); B-GlcNAc; C-mannose; D-galactose; E-sialic acid; F-fucose. Presented graphs were recalculated and plotted from multiple SPR data series.

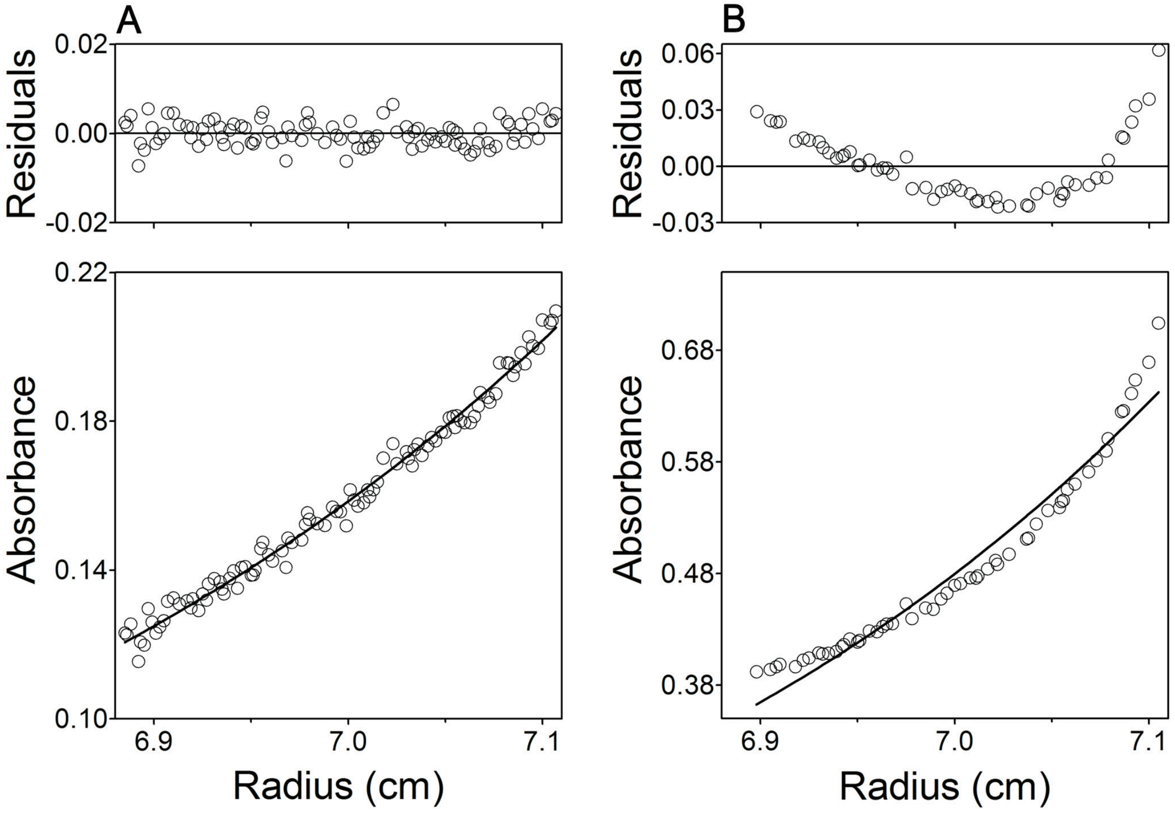

Figure S2. Sedimentation equilibrium studies of GRFN-1. Sedimentation equilibrium profiles were obtained for 36000 rpm (A) and 7000 rpm (B) at 20 °C. For experimental details see Materials & Methods section.

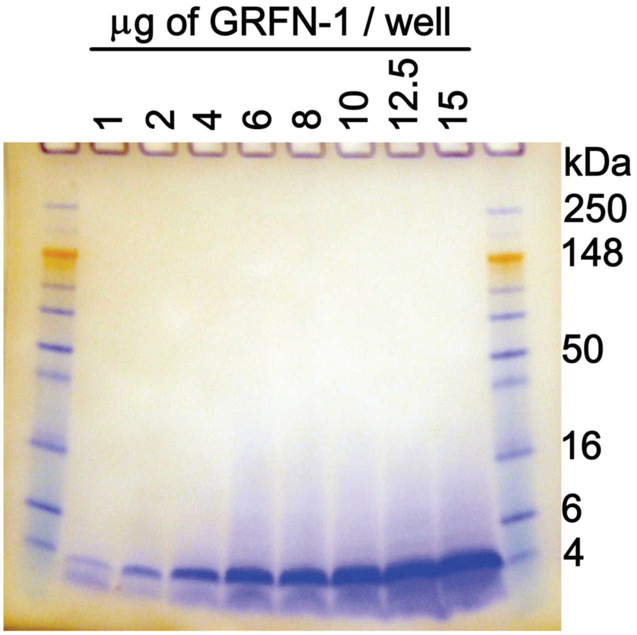

Figure S3. SDS-gel electrophoretic experiment for GRFN-1. Electrophoresis was performed in non-reductive conditions, using 10-20% polyacrylamide gradient gel which was subsequently stained with Colloidal Blue Staining kit (Invitrogen, Carlsbad, CA).

Table S1. Read-out of relative concentrations of inflammatory cytokines from the medium of primary vaginal epithelial cells (VEC) treated with various concentrations of GRFN-1.

GRFN-1	Observed concentration±SEM									
	**Hu IL-1rα**	**Hu IL-8**	**Hu IL-17**	**Hu GM-CSF**	**Hu VEGF**	**Hu CTACK**	**Hu GROα**	**Hu IL-1α**	**Hu IL-2Rα**	**Hu IL-3**
**100 μM**	820.6±237.4	33.7±23.1	4.6±0.0	23.3±5.3	366.8±48.8	9.7±1.9	26.6±3.4	25.7±10.9	6.0±0.7	12.8±1.4
**10 μM**	370.6±54.1	22.7±20.0	4.2±2.4	32.5±14.7	316.6±1.3	7.2±0.2	22.1±3.5	13.6±1.9	4.8±0.2	12.3±1.2
**1 μM**	203.3±61.5	77.7±0.0	7.1±1.8	38.4±10.8	191.2±113.1	11.2±2.5	24.3±5.7	11.4±3.6	7.9±2.1	13.4±0.4
**Vehicle**	76.0±66.8	19.8±0.0	OOR	23.5±7.9	28.6±26.2	7.6±0.2	21.8±1.7	21.1±1.1	5.2±0.2	10.2±0.4
	**Hu IL-12p40**	**Hu IL-16**	**Hu IL-18**	**Hu LIF**	**Hu MCP-3**	**Hu M-CSF**	**Hu MIF**	**Hu MIG**	**Hu β-NGF**	**Hu SCF**
**100 μM**	29.8±2.5	4.6±0.0	7.0±1.4	10.5±0.2	7.0±0.2	13.8±0.7	3241.9±1514.1	1.9±0.2	0.7±0.0	6.8±0.4
**10 μM**	21.6±0.9	4.0±0.0	2.7±2.2	8.7±0.4	5.7±0.2	12.1±0.6	1459.4±433.7	1.4±0.0	0.6±0.0	5.6±0.2
**1 μM**	30.1±5.9	4.7±1.2	0.8±0.1	10.5±1.8	8.0±1.6	13.9±2.6	2460.7±514.8	2.6±0.5	0.9±0.3	5.5±0.1
**Vehicle**	25.7±1.5	4.4±0.1	1.2±0.1	8.3±0.7	6.0±0.3	9.8±0.4	447.5±99.5	1.7±0.1	0.6±0.0	5.3±0.2
	**Hu SDF-1α**	**Hu TNF-β**	**Hu TRAIL**	**Hu HGF**	**Hu IFN-α2**					
**100 μM**	26.5±0.4	3.0±0.7	19.8±0.9	5.2±0.6	8.8±0.7					
**10 μM**	22.5±0.0	2.3±0.0	14.0±1.6	4.6±0.2	6.1±0.6					
**1 μM**	34.6±13.7	2.6±0.4	30.7±8.0	6.6±1.7	8.0±0.8					
**Vehicle**	22.5±0.0	2.4±0.1	17.6±0.3	4.1±0.0	5.8±0.0					

OOR-out of range

Table S2. Read-out of relative concentrations of inflammatory cytokines from the medium of human peripheral blood mononuclear cells (PBMC) treated with various concentrations of GRFN-1.

GRFN-1	Observed concentration±SEM									
	**Hu IL-2**	**Hu IL-5**	**Hu IL-8**	**Hu IL-10**	**Hu IL-13**	**Hu G-CSF**	**Hu GM-CSF**	**Hu IP-10**	**Hu MIP-1α**	**Hu RANTES**
**100 μM**	7732.5±**370.2**	137.0±51.0	2.6±0.0	9.3±0.0	580.1±168.8	24.9±0.0	7.2±4.3	33.2±0.0	52.4±4.5	161.5±108.7
**10 μM**	6165.9±**302.3**	92.2±4.3	6.4±0.0	3.6±1.7	629.2±13.9	23.9±13.5	4.6±0.0	34.5±17.0	49.9±7.8	251.6±8.9
**1 μM**	8058.7±**0.0**	181.8±39.5	8.8±3.9	12.1±10.2	987.9±140.4	52.7±20.2	26.5±14.1	83.0±8.4	113.1±24.7	309.6±39.4
**Vehicle**	9659.6±**0.0**	182.8±12.6	13.4±12.2	50.9±19.6	989.0±207.0	OOR	38.9±18.7	29.8±0.0	70.0±15.5	49.5±1.2
	**Hu VEGF**	**Hu IFN-γ**	**Hu MIP-1β**	**Hu CTACK**	**Hu GROα**	**Hu IL-1α**	**Hu IL-2Rα**	**Hu IL-3**	**Hu IL-12p40**	**Hu IL-16**
**100 μM**	7.3±3.5	1.1±0.0	333.0±51.4	20.8±1.9	22.8±1.4	2.0±0.1	27.4±2.0	23.0±0.8	32.8±2.3	1234.0±600.3
**10 μM**	6.8±0.8	1.1±0.0	338.5±52.4	22.8±0.0	26.3±0.6	2.0±0.0	29.5±0.4	25.2±1.6	29.5±0.9	2358.7±124.2
**1 μM**	20.4±6.7	37.5±20.4	658.3±118.1	18.4±1.0	23.2±1.2	1.9±0.1	30.9±1.8	18.4±0.4	22.7±1.1	2228.2±171.3
**Vehicle**	39.1±16.6	54.4±0.0	541.1±169.7	12.1±0.0	17.4±0.5	1.7±0.0	21.3±1.0	15.6±0.8	24.9±1.1	259.2±7.3
	**Hu IL-18**	**Hu LIF**	**Hu MCP-3**	**Hu M-CSF**	**Hu MIF**	**Hu MIG**	**Hu β-NGF**	**Hu SCF**	**Hu SCGF-β**	**Hu SDF-1α**
**100 μM**	1.4±0.2	10.8±2.4	8.9±0.7	14.9±0.5	4003.9±1442.0	5.9±0.8	1.5±0.2	7.8±0.8	750.1±415.6	75.4±8.4
**10 μM**	1.7±0.0	8.8±0.3	10.2±0.1	15.8±0.8	5968.3±546.1	7.8±0.4	1.8±0.1	8.8±0.2	446.2±89.5	89.5±5.7
**1 μM**	1.1±0.0	7.5±0.1	9.3±0.2	13.6±0.0	3991.9±253.1	7.5±1.4	1.4±0.1	6.7±0.3	105.6±2.9	62.1±4.1
**Vehicle**	0.6±0.0	9.1±0.4	6.1±0.2	12.9±0.5	1000.8±69.9	4.0±0.0	0.9±0.0	8.4±0.0	1032.9±62.0	46.2±0.7
	**Hu TNF-β**	**Hu TRAIL**	**Hu HGF**	**Hu IFN-α2**						
**100 μM**	49.3±17.9	54.6±1.4	7.8±0.5	44.1±8.4						
**10 μM**	45.7±9.3	72.0±3.5	8.6±0.0	57.1±2.4						
**1 μM**	65.2±8.4	82.5±14.0	7.6±0.7	48.8±0.1						
**Vehicle**	62.9±4.9	38.5±1.5	5.3±0.5	19.4±1.5						

OOR-out of range
